# On the link between attentional search and the oculomotor system: Is preattentive search restricted to the range of eye movements?

**DOI:** 10.3758/s13414-019-01949-4

**Published:** 2020-01-15

**Authors:** Soazig Casteau, Daniel T. Smith

**Affiliations:** Department of Psychology, Durham University, Durham, UK

**Keywords:** Attention, Covert visual search, Preattentive search, Eye movement, Oculomotor control

## Abstract

It has been proposed that covert visual search can be fast, efficient, and stimulus driven, particularly when the target is defined by a salient single feature, or slow, inefficient, and effortful when the target is defined by a nonsalient conjunction of features. This distinction between fast, stimulus-driven orienting and slow, effortful orienting can be related to the distinction between exogenous spatial attention and endogenous spatial attention. Several studies have shown that exogenous, covert orienting is limited to the range of saccadic eye movements, whereas covert endogenous orienting is independent of the range of saccadic eye movements. The current study examined whether covert visual search is affected in a similar way. Experiment 1 showed that covert visual search for feature singletons was impaired when stimuli were presented beyond the range of saccadic eye movements, whereas conjunction search was unaffected by array position. Experiment 2 replicated and extended this effect by measuring search times at 6 eccentricities. The impairment in covert feature search emerged only when stimuli crossed the effective oculomotor range and remained stable for locations further into the periphery, ruling out the possibility that the results of Experiment 1 were due to a failure to fully compensate for the effects of cortical magnification. The findings are interpreted in terms of biased competition and oculomotor theories of spatial attention. It is concluded that, as with covert exogenous orienting, biological constraints on overt orienting in the oculomotor system constrain covert, preattentive search.

**Electronic supplementary material:**

The online version of this article (10.3758/s13414-019-01949-4) contains supplementary material, which is available to authorized users.

Visual search describes the cognitive process of locating target objects among distracters. According to Treisman’s seminal feature integration theory (Treisman & Gelade, 1980), search is composed of two stages: a preattentive stage, in which the entire scene is processed in parallel to identify basic visual features such as colour and orientation, and an attentive stage, in which spatial attention is used to bind spatially congruent features into more complex objects. In this view, targets defined by a single feature can be quickly and efficiently identified during preattentive search, and the search time is unaffected by the number of distractors. In contrast, identification of targets defined by a conjunction of features requires an inefficient and effortful serial movement of attention to different spatial locations until the correct combination of features is found. More recent theories, such as Wolfe’s guided search (Wolfe, 1994), argue that some conjunction searches can be efficient if basic visual features can be used to guide attention to a subset of relevant stimulus conjunctions, but retain the core idea that some searches are fast, automatic, and stimulus driven, whereas others are slow, effortful, and goal directed.

This distinction between fast, efficient stimulus driven orienting and slow, inefficient goal directed orienting echoes the distinction drawn by Posner, Snyder, and Davidson (1980) between exogenous spatial attention and endogenous spatial attention. In Posner’s studies, participants were shown a cue comprising a salient peripheral luminance flicker or a centrally presented arrow. The peripheral cue elicited rapid, reflexive, and transient orienting, whereas the central arrow elicited a slower, but consciously controlled and sustained, shift of attention. These exogenous and endogenous shifts of attention could be instantiated overtly as movements of the eye, or covertly, such that the spotlight of attention was oriented independently of gaze direction. A major debate subsequently developed around the precise relationship between the mechanisms that control eye movements and the mechanisms of covert spatial attention.

One influential view was Klein’s oculomotor readiness hypothesis (OMRH; Klein 1980), which proposed that covert orienting of attention was caused by the programming of an eye movement. Although Klein and colleague rejected OMRH on the basis that there was no attentional facilitation at the location of planned but unexecuted eye movement (Hunt & Kingstone, 2003; Klein & Pontefract, 1994), subsequent studies demonstrated that eye movements were always preceded by a mandatory shift of attention to the saccade goal, consistent with the idea that saccade planning is sufficient to orient attention (Deubel & Schneider, 1996; Hoffman & Subramaniam, 1995; Shepherd, Findlay, & Hockey, 1986). The idea of a tight coupling between attention and oculomotor control was subsequently developed by Rizzolatti, Riggio, Dascola, and Umilta (1987) as the premotor theory of attention. The strongest form of premotor theory argued that activation of the oculomotor system is both necessary and sufficient for covert orienting of attention (Rizzolatti, Riggio, & Sheliga, 1994). Many studies seemed to offer convincing evidence that the neural systems used for the control of spatial attention and the control of eye movements were largely overlapping (Corbetta et al., 1998; de Haan, Morgan, & Rorden, 2008; Fairhall, Indovina, Driver, & Macaluso, 2009; Gitelman, Parrish, Friston, & Mesulam, 2002; Perry & Zeki, 2000), and premotor theory became one of the dominant models of spatial attention.

However, Premotor Theory has not gone unchallenged. For example, Deubel and Schneider (1996) argued that the presaccadic allocation of attention to a saccade endpoint occurred because attentional selection of a spatial location is required in order to define the spatial coordinates of the saccade goal. In their visual attention model, covert allocation is necessary for the orienting of attention, which is the opposite of the claim made by premotor theory. Furthermore, Born, Mottet, and Kerzel (2014) have elegantly demonstrated that saccade programming only elicits a presaccadic shift of attention when the saccade is executed, suggesting that saccade programming alone is insufficient to produce covert orienting, and Dunne, Ellison, and Smith (2015) reported that it was possible to use instrumental conditioning to change the metrics of saccade programs without disrupting covert orienting. In related work, Belopolsky and Theeuwes (2009a, 2009b, 2012) showed that participants could sustain attention at a location without activating a saccade plan (see also MacLean, Klein, & Hilchey, 2015). This echoes the assumption firstly made by Klein and Pontefract (1994) that premotor theory might still be tenable for shifting, but not for sustaining, attention.

We also challenged premotor theory in a series of studies that demonstrated that the ability to plan and execute saccadic eye movement was not required to produce endogenous, covert orienting of attention. For example, we reported a patient with congenital ophthalmoplegia (paralysis of the eyes) who could not make saccadic eye movements (Smith, Rorden, & Jackson, 2004). This patient had a deficit of exogenous covert orienting, but no problem with endogenous orienting. Other studies have reported similar patterns of impaired exogenous orienting, but preserved endogenous orienting in other patients with ophthalmoplegias caused by Duane's syndrome, which is associated with problems with abductive eye movements (Gabay, Henik, & Gradstein, 2010) and progressive supranuclear palsy (PSP), which is characterized by vertical gaze paralysis (Rafal, Posner, Friedman, Inhoff, & Bernstein, 1988). Patients with PSP, who present with impaired exogenous orienting, but relatively preserved vertical endogenous attentional orienting (Rafal et al., 1988), also have impaired spatial working memory along the vertical axis (Smith & Archibald, 2018), which may partially be caused by problems attending to and encoding the stimuli. This neuropsychological dissociation between oculomotor control and endogenous attention seems hard to reconcile with the idea of a strict coupling between saccade control and covert attention.

One might be cautious in rejecting premotor theory based solely on small studies of neuropsychological patients. However, these effects have been replicated in healthy participants using an eye-abduction paradigm (Craighero, Nascimben, & Fadiga, 2004) to experimentally constrain eye movements. In these studies, the stimuli were viewed monocularly with the eye abducted from the midline at an angle of 40 degrees. This places the stimuli at the edge of the effective oculomotor range (EOMR), which is the range locations that can be reached with a saccadic eye movement that does not also require a head movement (Guitton & Volle, 1987). This manipulation reliably elicits deficits of exogenous orienting (Morgan, Ball, & Smith, 2014; Smith, Schenk, & Rorden, 2012) and visuospatial working memory (Ball, Pearson, & Smith, 2013; Pearson, Ball, & Smith, 2014) while having little effect on endogenous orienting (Smith, Ball, & Ellison 2014; Smith et al., 2012; although see Craighero et al., 2004). On the basis of these studies we argued that premotor theory is only valid for exogenous orienting, a view that is consistent with a growing body of other experimental and neuropsychological evidence (Casteau & Smith, 2018; Smith et al., 2014; Smith & Schenk, 2012).

Exogenous shifts of attention and efficient preattentive search rely on the same rapid, stimulus-driven mechanisms that allow the selection of salient spatial location on the saliency map. In the paradigmatic task used to measure exogenous attention (the peripheral cueing task; Posner, 1980) salience is driven by the appearance of a transient cue, whereas in the case of efficient search, the salience is driven by the uniqueness of the target relative to the surrounding distractors. At a behavioural level, both exogenous attention and efficient preattentive search are rapid and automatic but short lived and overruled by a long-lasting inhibitory effect at the location of the salient event (Klein, 2000), known as inhibition of return (Klein & MacInnes, 1999; Posner, Rafal, Choate, & Vaughan, 1985), and numerous studies have shown that attention capture by peripheral transient cues engages the same cognitive processes as the capture by salient singletons in preattentive feature search task (e.g., Theeuwes, Kramer, Hahn, Irwin, & Zelinsky, 1999).

If premotor theory is correct for exogenous orienting, and if ‘preattentive search’ and exogenous orienting reflect the same underlying cognitive mechanism, then we can make a clear prediction about the role of the eye-movement system in preattentive search. Specifically, preattentive search should be dependent on the activation of the oculomotor system. Consistent with this idea, eye-abduction interferes with feature search, such that covert visual  search is delayed by ~60 ms when the target is beyond the EOMR (Smith, Ball, Ellison, & Schenk, 2010). This delay is not observed when participants are required to perform a conjunction search (Smith et al., 2014), consistent with our and Klein’s conclusion that covert, endogenous orienting is independent of saccade control. Together, these data seem to suggest that the oculomotor system is critical for fast, efficient, stimulus-driven processing, but is rather less important for inefficient, goal-directed search

However, a couple of factors suggest that we might be reluctant to conclude that preattentive search is strictly limited to the range of eye movements. Firstly, eye abduction requires participants to adopt a very unusual viewing position, and this may not reflect how visual search operates in the real world. Secondly, studies using eye abduction and patients with ophthalmoplegia may confound the inability to make eye movements with the disruption to proprioceptive signals about eye position. This may be problematic, because Balslev and colleagues have shown that oculoproprioception plays an important role in covert spatial attention (Balslev, Newman, & Knox, 2012). Finally, it is not clear whether disrupting the oculomotor system simply slows parallel processing during preattentive search, or reduces the efficiency of preattentive search to the extent that participants must engage serial search processes.

To address these issues, we took advantage of the fact that the visual field is considerably larger than the effective oculomotor range. Specifically, the visual field extends up to 90 degrees in the temporal visual field (Niederhauser & Mojon, 2002), whereas the EOMR is ~40 degrees to either side of fixation (Guitton & Volle, 1987). This discrepancy means stimuli can be presented that are visible, but not accessible, by a saccadic eye movement. We have previously used this approach to examine the role of oculomotor control in attention using the Posner cueing task (Casteau & Smith, 2018). In this study, the EOMR was established for each participant and the stimuli positions individualized to ensure they were within or beyond each individual’s EOMR. The critical finding was that exogenous, covert orienting was abolished beyond the EOMR, whereas endogenous, covert orienting was preserved, exactly as what had previously been observed in patients with ophthalmoplegia and in studies using eye abduction. Following the same logic, in the current tasks we measured participants EOMR and asked them to complete conjunction and feature search tasks with stimuli presented either within or beyond their EOMR. If our proposal that exogenous covert attention is dependent on the oculomotor system is correct, covert feature search should be impaired for arrays presented beyond the EOMR, whereas covert conjunction search should be unaffected by array location.

## Materials and methods

In both experiments, a search array composed of five or eight items was presented at locations within or beyond the effective oculomotor range (EOMR). The type of search was manipulated so that it was either a feature search (*colour* and *orientation* in Experiment 1, *colour* in Experiment 2) or a conjunction search task (Experiment 1 and Experiment 2). The main differences between Experiments 1 and 2 were as follows: Experiment 1 used two array locations whereas Experiment 2 used six locations; Experiment 1 compared colour and orientation feature search tasks to conjunction search, whereas Experiment 2 compared only colour feature search to conjunction search. In Experiment 1, participants knew the identity of the feature search targets, whereas in Experiment 2 they did not; and Experiment 1 had 25% no-target trials, whereas Experiment 2 had 50% no-target trials.

Before each of the two experiments, we conducted power analyses to establish the minimum sample sizes required to observe a difference in reaction times between the different array locations. For Experiment 1, we based the analysis on the data from Smith et al. (2014). They observed an increase of 62.5 ms (*SD* = 51 ms) in search time in the feature search task when the target appeared beyond the EOMR compared with when it appeared within the EOMR. The analysis estimated that at least 13 participants would be needed to obtain a statistical power at the recommended .95 level (Cohen, 1988; *d*_z_ = 1.22). For Experiment 2, we establish the minimum sample size required to observe a significant difference between the within the EOMR and beyond the EOMR conditions in the feature colour search task based on the data from Experiment 1. The analysis estimated that at least nine participants would be needed to obtain a statistical power at the recommended .95 level for a set size of three (five items) (*M*_diff_ = 47 ms, *SD*_diff_ = 46, *d*_z_ = 1.01) and eight participants for a set size of six (eight items) (*M*_diff_ = 67 ms, *SD*_diff_ = 78, *d*_z_ = .85).

### Stimulus material

**Fig. 1:**
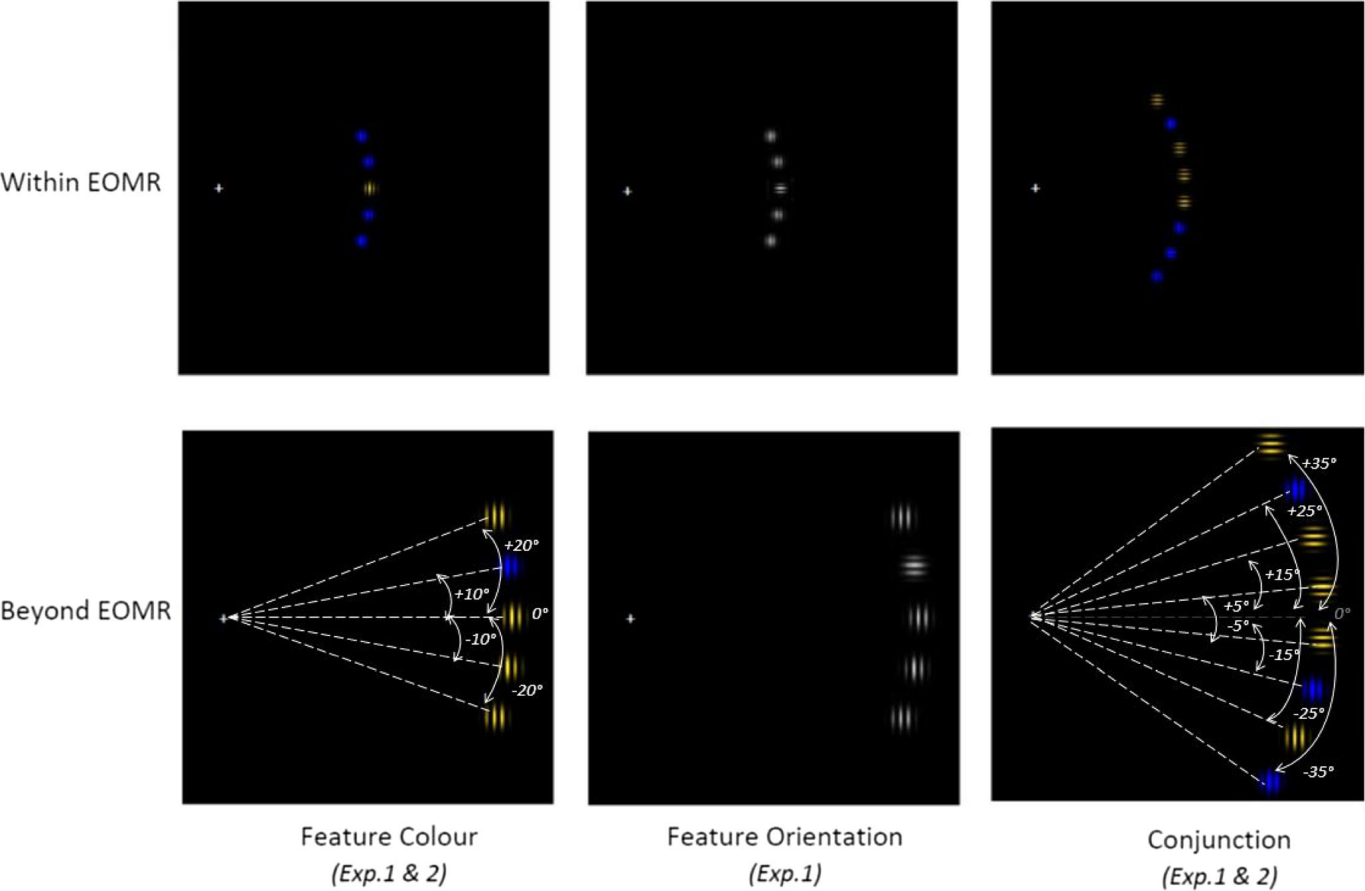
Schematic of stimulus material (not to scale). The upper row shows search arrays presented within the oculomotor range (EOMR), the lower row shows arrays presented beyond the oculomotor range. The colour search task (Experiments 1 & 2) is shown on the leftmost panels*,* the orientation search task (Experiment 1) in the middle panels, and the conjunction search task (Experiments 1 & 2) in the rightmost panels. Dotted lines and arrows indicate the stimuli location (not shown in the experiment)

The search array consisted of a fixation cross (“+” sign, .2°) and circle shapes (.4°) filled with black/grey grating (Experiment 1 feature orientation search task) or black/yellow and black/blue grating (Experiment 1 & Experiment 2 feature colour and conjunction search task) presented on a black background. Within each block of trials, the target could randomly appear at one of three or six possible locations within the array. The two most extreme locations were never target locations. For a set size of three (five items), circles were presented on the horizontal axis (0°) at 10° and 20° above and below the horizontal axis, for a set size of six (eight items), circles were presented at 5°/15°/25° and 35° above and below the horizontal axis (see Fig. 1). In Experiment 1, the visual array could be presented at two locations, either within the range of eye movement (i.e., within the EOMR) or outside the range of eye movements (i.e., beyond the EOMR), with the precise location depending on the individual participants EOMR. In Experiment 2, the visual array was presented at six different locations ranging from 29° to 44° by steps of 3°.

In the feature colour search (Experiment 1 & Experiment 2), the target could be a black/yellow grating among black/blue gratings, or a black/blue grating among black/yellow gratings. In the feature orientation search (Experiment 1), the distracter items were all oriented in the same direction (vertically or horizontally), and the target was orientated perpendicularly to the distracters. In the conjunction search (Experiment 1 & Experiment 2), participants had to search for both a specific colour and orientation—for example, search for vertically oriented yellow grating presented among vertical, blue gratings and horizontal, yellow gratings (see Fig. 1). Each participant was presented with one colour/orientation combination, each combination was randomly assigned and counterbalanced across participants (i.e., seven and eight participants were presented with the vertical yellow target, and six and seven participants with the horizontal blue target, in Experiment 1 and Experiment 2, respectively).

Stimuli were scaled in accordance with the cortical magnification equation of Rovamo and Virsu (1979), using the following formula:

1 $$ s\times \ast \left(\left(1+.29\times \ast e\right)+\left(.000012\times \ast {e}^3\right)\right), $$

where *s* was the circle shape size and *e* was the array eccentricity. In Experiment 1, visual search array eccentricities ranged between 12° and 20° for the within EOMR condition and between 37° and 44° for the beyond EOMR condition.

### Apparatus

Stimuli were generated using PsychoPy (Peirce, 2009) and saved as a JPEG. They were displayed on a LED monitor (BenQ) driven by an NVIDIA GeForce GTX 750 Ti graphics board at a refresh rate of 60 Hz. The resolution of the monitor was set at 2560 × 1440 pixels, which corresponded to physical dimensions of 708-mm wide by 398-mm high. At a viewing distance of 30 cm, the display occupied a viewing area of 99° horizontally and 67° vertically. Fixation was monitored using an EyeLink II (SR Research Ltd., Mississauga, Ontario, Canada) sampling at 500 Hz in pupil-only mode. Manual responses were collected using a two-button box set with a TTL trigger.

### Procedure

The experiments were run at Durham University, UK. They lasted about 1.5 to 2.5 hours, as participants completed the experiments at their own pace. All participants gave informed consent to participate in the study, which was run in accordance with the Durham University Department of Psychology Research Ethics Committee and were conducted in accordance with the British Psychological Society code of ethics.

The room was dimly illuminated by a diffuse indirect light source. The participant was seated in an adjustable chair in front of a computer screen, and a chin rest was used to minimize head movements. Each block of trials began with calibration of the EyeLink II. If the correlation between the actual and the estimated eye location was satisfactory, the block of trials began; otherwise, another calibration phase was initiated.

Participants performed the task monocularly with the dominant eye, the nondominant eye being patched. Eye dominance was assessed using a ‘framing method’. Each participant was seated ±2 m away from the experimenter and was asked to fixate on the nose of the experimenter. They were then asked to extend their arms and bring their hands together in front of the eyes, leaving only a small gap through which the experimenter could see one of the participants’ eyes. The visible eye was recorded as dominant.

Prior to starting each experiment, we measured each participant’s EOMR for the temporal visual field (see Supplementary Material). This allowed us to calculate visual search array eccentricities for each participant individually for Experiment 1 and map the array location and the EOMR effect for Experiment 2.

Each trial began with the presentation of a fixation cross for 1000 ms, followed by the search array. Participants were asked to stay fixated on the central cross during the duration of the trial. They were then instructed to respond as quickly as possible by pressing one of two buttons to indicate whether the target was present or absent (target-detection task). The search array remained visible until the manual response was made. This was followed by the presentation of a black screen for 1000 ms; after this delay, the next trial began.

### Data selection and analysis

Data were filtered to remove trials in which (1) participants made a saccade of an amplitude of more than 2° and (2) response was faster than 150 ms (anticipation). Statistical analyses were performed using R Version 3.5.0 (R Core Team, 2018), power analyses were performed using the package ‘pwr’ (Champely et al., 2018), ANOVAs were conducted using the ‘ez’ package (Lawrence, 2015), data visualization was performed with the package ‘ggplot2’ (Wickham, 2009), planned contrasts were performed using the package ‘emmeans’ (Lenth et al., 2019). In case of a violation of the assumption of sphericity (Mauchly’s test of sphericity, Mauchly, 1940), we used the Greenhouse–Geisser correction to report the corrected degrees of freedom and *p* values. Significant effects were explored using Bonferroni-corrected paired-samples *t* tests, where the baseline alpha value of .05 was divided by the number of comparisons.

## Experiment 1

### Method

#### Participants

Thirteen participants total (eight females, two left-handed, one left-eyed, *M*_age_ = 28 years) reported having normal vision and were unaware of the purpose of the experiment.

#### Design

Each participant performed all three tasks (feature colour, feature orientation, and conjunction), the tasks order was randomized between participants. Data were subject to a 3 × 2 × 2 ANOVA, with within-subjects factors of (1) search task with three levels (feature colour, feature orientation, or conjunction), (2) set size with two levels (3 vs. 6), and array location with two levels (within vs. beyond). There were 60 repetitions per combination of each factor level, which resulted in a total of 720 trials (540 trials target present and 180 trials target absent). All participants started each search task with one block of 20 practice trials followed by six blocks of 120 experimental trials (two blocs for feature colour, two blocks for feature orientation, and two blocks for conjunction search).

### Results

One participant showed an average accuracy of 59% (colour: 62%, orientation: 61%, conjunction: 56%) and was excluded from the analysis. Data from the remaining 12 participants were filtered, and data were excluded if they did not meet the above criteria (see Materials and methods section). On average, there were 3.1% rejected trials in feature colour, 2.8% in feature orientation, and 7.6% in conjunction, corresponding to an average of 5.15% for criteria (1) and .05% criteria (2) across the three different search tasks. This led to an average number of 232 trials in feature colour, 237 trials in the feature orientation, and 221 trials in the conjunction search task.

#### Manual reaction times

**Fig. 2:**
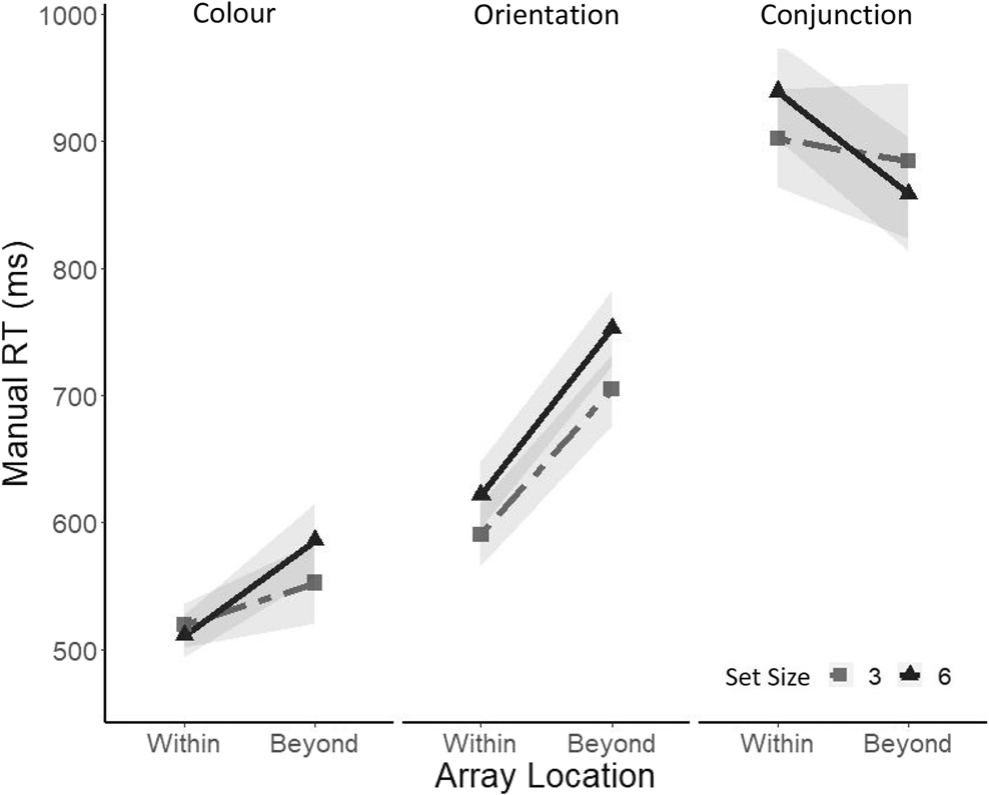
Median reaction times for correct responses on target-present trials as a function of array location, separately for a set size of three (light grey) and a set size of six (dark grey) and for each search task (colour: leftmost panel; orientation: middle panel; conjunction: rightmost panel). Shadows represent ±1 *SEM*

Median manual reaction times (RTs) for target-present trials and correct responses were analyzed. The ANOVA revealed a main effect of search task, *F*(2, 22) = 44.84, *p* < .0001, ηp² = .80. As can been seen in Fig. 2, median RTs were slower in the conjunction task (*Mdn* = 896 ms) compared with the feature orientation (*Mdn* = 667 ms) and the feature colour task (*Mdn* = 542 ms). We also observed a significant two-way interaction between search task and array location, *F*(2.3, 25.9) = 4.48, *p* < .05, ɛ = .55, ηp²  = .29. ANOVAs with a factor of array location at each level of search task revealed a main effect of array location in the feature colour task (within EOMR= 514 ms, beyond EOMR = 572 ms), *F*(1, 11) = 4.97, *p* < .05, ηp² = .31, and the feature orientation task (within EOMR = 611 ms, beyond EOMR = 738 ms), *F*(1, 11) = 20.3, *p* < .0001, ηp² = .65. However, there was no effect of array location in the *C*onjunction task, *F*(1, 11) = .015.

#### Manual reaction time: target absent

**Fig. 3:**
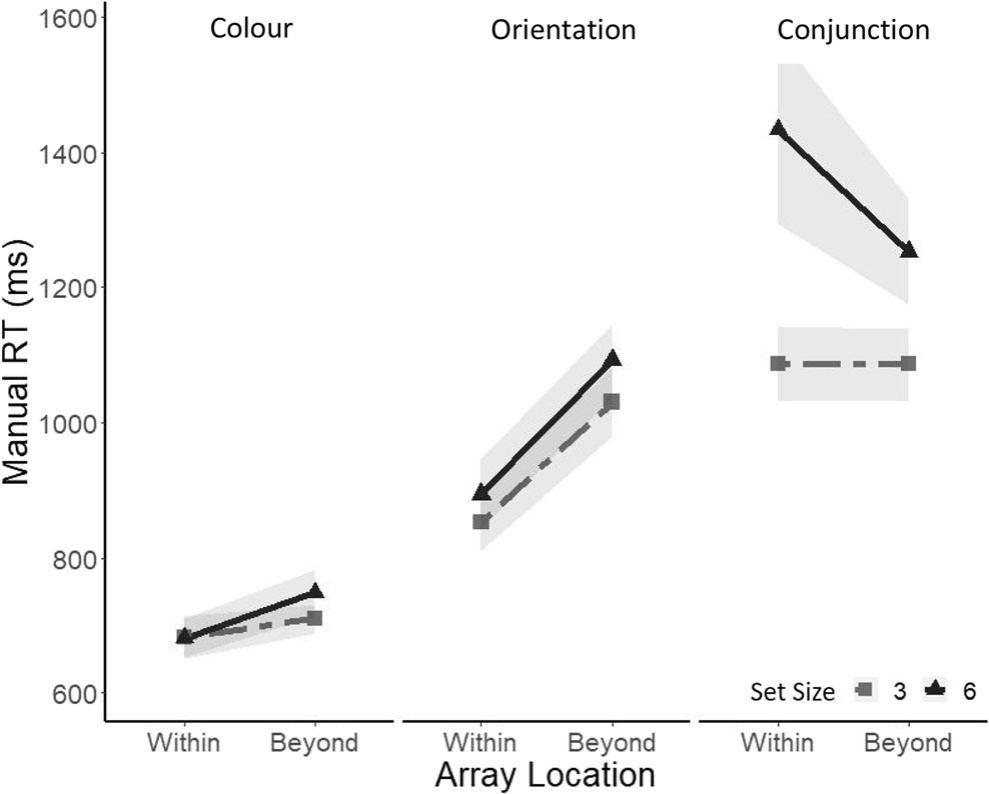
Median reaction times for correct responses on target-absent trials as a function of array location, separately for a set size of three (light grey) and a set size of six (dark grey) and for each search task (colour: leftmost panel; orientation: middle panel; conjunction: rightmost panel). Shadows represent ±1 *SEM*

Median RTs for correct rejections on target-absent trials are reported in Fig. 3. An ANOVA performed on median RTs revealed a main effect of search task, *F*(1.1, 12.1) = 37.31, *p* < .0001, ɛ = .75, ηp² = .77, such that participants were slower in the conjunction task (*Mdn* = 1,215 ms) compared with the feature orientation (*Mdn* = 967 ms) and the feature colour (*Mdn* = 705 ms). We also observed a significant effect of set size, *F*(1, 11) = 14.12, *p* < .001, ηp^2^ = .56, as participants were slower for the six items (*Mdn* = 1,017 ms) compared with the three-items set size (*Mdn* = 908 ms). We also observed a significant interaction between search task and set size, *F*(1.85, 20.35) = 5.84, ɛ = .65, *p* < .001, ηp^2^ = .35, as well as an interaction between array location and search task, *F*(2, 22) = 3.93, *p* < .05, ηp^2^ = .26.

The Search Task × Set Size interaction was explored with one-way ANOVAs conducted at each level of the search task. This analysis revealed a significant effect of set size in the conjunction search (Set Size 3: 1,087 ms; Set Size 6: 1,343 ms), *F*(1, 11) = 17.71, *p* < .01, ηp^2^ = .61; however, there was no effect of set size on the feature colour or feature orientation tasks, colour: *F*(1, 11) = .34; orientation: *F*(1, 11) = .39.

T tests, performed to explore the Search Task × Array Location interaction, revealed a significant effect of array location in the feature orientation task (within = 874 ms; beyond = 1,061 ms), *t*(11) = 3.37, *p* = .006. The effect of array location was not significant in both the feature colour task, *t*(11) = 1.56, *p* = .14, and the conjunction task, *t*(11) = .87, *p* = .4.

#### Response accuracy

As can be seen in Table 1, where both average hit rates and false alarms (FA) are presented, participants performed on average better in the feature search tasks (colour: .98; orientation: .95) compared with the conjunction search task (.86). Participants were also more prone to FA in the conjunction task (.19) compared with the feature orientation (.13) and feature colour (.04) tasks.

**Table 1:** Average rates and *standard deviations* for hit, false alarm (FA), and *d* prime (*d'*) separately for each search task, array position, and set size

	Feature colour				Feature orientation				Conjunction			
	Below 3	Beyond 3	Below 6	Beyond 6	Below 3	Beyond 3	Below 6	Beyond 6	Below 3	Beyond 3	Below 6	Beyond 6
Hit	.99 *.02*	.97 *02*	.98 *.02*	.98 *.02*	.94 *.02*	.96 *.01*	.94 *.01*	.96 .*01*	.91 *.02*	.85 *.06*	.87 *.03*	.81 *.05*
FA	.03 *.02*	.06 *.03*	.03 *.02*	.03 *.02*	.14 *.03*	.11 *.03*	.13 *.03*	.14 *.02*	.07 *.02*	.24 *.05*	.19 *0.4*	.24 *.04*
d'	3.7	3.6	4.1	4.1	3.4	3.4	3.7	3.6	3.1	2.7	3.1	2.5

Perceptual sensitivity was computed (*d* prime [*d’*]), an ANOVA performed on *d* prime values showed a main effect of search task, *F*(2, 22) = 24.19, *p* < 0.001, ηp^2^ = 0.68, as well as a main effect of set size, *F*(1, 11) = 4.95, *p* < 0.05, ηp^2^ = .31. There were also significant interactions between search task and set size, *F*(2, 22) = 7.76, *p* < .01, ηp^2^ = 0.41, and between search task and array location, *F*(2, 22) = 5.04, *p* < 0.05, ηp^2^ = .31. A one-way ANOVA with a factor of set size performed separately for each search task revealed that participants had significantly better sensitivity for the set size of six items compared with the set size of three items in the colour search task (three items = 3.65; six items = 4.09), *F*(1, 11) = 25.56, *p* <.001*,* =.45 ) and the orientation search task (3 items = 3.38, 6 items = 3.65, *F*(1,11) = 5.74, *p* < 0.05, ηp^2^ = 0.34), but not in the conjunction search task (*F*(1, 11) = 0.56). T tests were then performed to explore the interaction between search task and array location and revealed no significant effect, colour: *t*(11) = .16, *p* = .87; orientation: *t*(11) = .15, *p* = .88; conjunction: *t*(11) = 2.16, *p* = .054.

### Discussion

Experiment 1 examined the influence of the EOMR on covert visual search. When the target was present, we observed that search for colour and orientation singletons was significantly slower when search arrays were presented beyond the EOMR, whereas conjunction search was unaffected by the EOMR. In the target-absent trials, we observed an interaction between the search task and the array location, such that participants tended to take longer to correctly reject trials when the array appeared beyond the EOMR in the feature search tasks, but tended be faster to correctly reject trials when the array appeared beyond the EOMR in the conjunction search task. There was also an interaction between search task and set size, such that the set-size effect was present in the conjunction search task but not the feature search tasks. The EOMR did not influence perceptual sensitivity during feature search, but there was a trend towards poorer sensitivity beyond EOMR during conjunction search. Given that participants also tended to have faster RTs beyond the EOMR in the conjunction search, this result may indicate a speed–accuracy trade-off in this condition

Overall, these data suggest that feature search tasks which engage efficient ‘preattentive’ search processes are delayed when the target appears at locations that cannot be the goal of a saccadic eye movement, whereas conjunction search tasks which require inefficient, ‘attentive’ processes are unaffected. On first inspection, these findings are consistent with our previous findings that exogenous, covert orienting is constrained by the EOMR (Casteau & Smith 2019; Smith et al., 2014; Smith et al., 2010; Smith et al., 2012), and the idea that reflexive attentional processes are tightly coupled with the oculomotor system, as predicted by oculomotor readiness theories of exogenous orienting (Casteau & Smith, 2019; Smith & Schenk, 2012). However, there are some caveats. Firstly, in both feature tasks, the participants were clearly instructed to search for one feature (e.g., ‘Look for the yellow target’ or ‘Look for the horizontally oriented target’). One issue with having an invariant target is that participants could potentially have implemented an attentional template to help detect the target, so one might argue that our feature search task was not a ‘pure’ measure of pre-attentive search. A previous study using eye abduction (Smith et al., 2010) used both a predictable and unpredictable feature singleton, and found that detection was delayed by similar magnitude when the target appeared beyond the EOMR in both conditions. Secondly, we observed the same effect of array location in the target-present and target-absent trials, but comparisons between these two conditions should be made with caution, as target was absent only in one fourth of the trials. We also observed a large number of FAs in the conjunction task, which may also result from the unbalanced number of target-present/target-absent trials. Thirdly, in the conjunction search task, we observed a significant set-size effect in the target-absent condition, but not the target-present condition, suggesting that some participants may have been using idiosyncratic strategies during this task. Finally, even though array locations were defined according to each individual’s EOMR, we cannot definitively rule out the possibility that the increase in RTs observed during feature search was due to an effect of eccentricity rather than to an effect related to constraints in the oculomotor system.

Experiment 2 was designed to address these issues by (1) presenting the search array at different locations in the periphery, (2) using a mixed target condition with unknown target identity, and (3) using an even number of target-absent/target-present trials.

## Experiment 2

### Method

#### Participants

Fifteen individuals participated (11 females, one left-handed, one left-eyed, *M*_age_ = 23 years), all reported having normal vision and were unaware of the purpose of the experiment.

#### Design

Each participant performed two search tasks (feature colour and conjunction). The task order was randomized between participants. In both tasks, the target appeared on 50% of the trials and with equal frequency at the different possible locations. In the feature colour search task, the target could either be yellow or blue so that participants did not know the target colour beforehand. Data were subject to a 2 × 2 × 6 ANOVA, with within-subjects factors of (1) search task with two levels (feature colour, conjunction), (2) set size with wo levels (3 vs. 6), and (3) array location with six levels (29°, 32°, 35°, 39°, 41°, and 44°). There were 50 repetitions per combination of each factor level (25 target present and 25 target absent), which resulted in a total of 1,200 trials (600 feature colour and 600 conjunction trials).

All participants started each search task with one block of 20 practice trials followed by 16 blocks of 75 experimental trials (eight blocks of feature and eight blocks of conjunction search).

### Results

Data were excluded if they did not meet the criteria specified in the Materials and methods section. Rejected trials represented 19% of the total number of trials across participants (14% in the feature colour and 24% in the conjunction search tasks). This led to an average number 456 trials in the conjunction task and an average of 516 trials in the feature colour task.

#### Manual reaction time: target present

Median manual reaction times for correct responses in target-present trials were analyzed first. An ANOVA revealed a significant main effect of search task, *F*(1, 14) = 49.76, *p* < .001, ηp^2^ = .78, such that participants were slower in the conjunction (*Mdn* = 939 ms) compared with the feature search task (*Mdn* = 591 ms). There was no effect of set size, *F*(1, 14) = 3.14, or array location, *F*(5, 70) = 1.89, as well as no significant interactions between all the three factors (all *F*s ≤ 3.14).

#### Manual reaction time: target absent

**Fig. 4:**
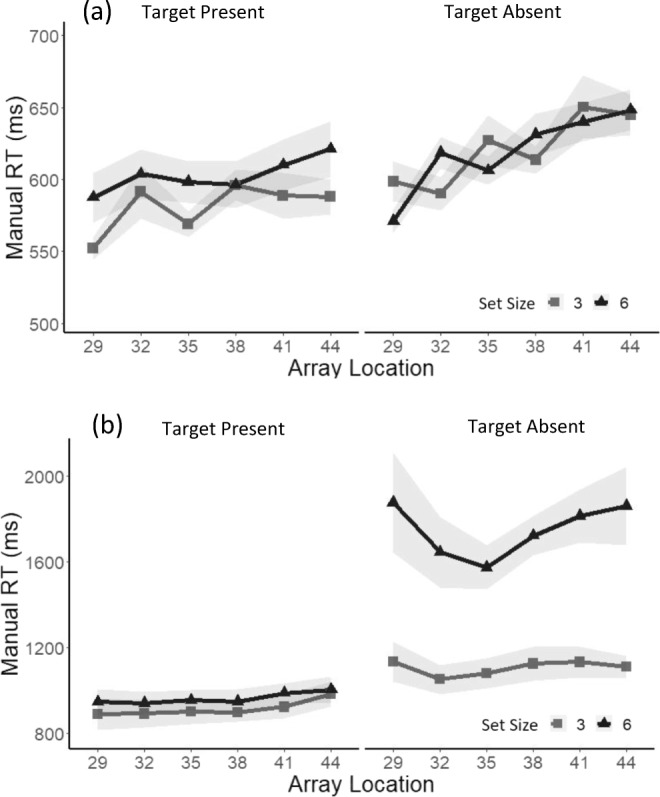
**a** Median reaction times in the feature search for correct responses in target-present trials (left) and target-absent trials (right) as a function of array location and separately for a set size of three (light grey) and a set size of six (dark grey). **b** Median reaction times in the conjunction search for correct responses in target-present trials (left) and target-absent trials (right) as a function of array location and separately for a set size of three (light grey) and a set size of six (dark grey). Shadows represent ±1 *SEM*

Median manual reaction times for correct responses in target-absent trials were then analyzed. An ANOVA revealed a main effect of search task (conjunction = 1,427 ms; feature: 620 ms), *F*(1, 14) = 67.48, *p* < .0001, ηp^2^ = .82, a main effect of set size (Set Size 3: 863 ms; Set Size 6: 1183 ms), *F*(1, 14) = 14.68, *p* < .001, ηp^2^ = .51 (see Fig. 4a–b) and a significant Set Size × Search Task interaction, *F*(1, 14) = 15.83, *p* < .0001, ηp^2^ = .53. A one-way ANOVA with a factor of set size performed separately for each search task revealed that participants were significantly slower for a set size of six (*Mdn* = 1679 ms) compared with a set size of three (*Mdn* = 1089), *F*(1, 14) = 15.38, *p* < .001, ηp^2^ = .52, in the conjunction search task, whereas there was so significant effect of set size in the feature colour search task, *F*(1, 14) = .004.

#### Manual reaction time: within and beyond the EOMR

The preceding analysis examined the effect of eccentricity on search times. There was no systematic effect of eccentricity on reaction time, thus ruling out the suggestion that the effect we observed in Experiment 1 could be accounted for solely by the eccentricity of the stimuli. However, unlike with Experiment 1, this analysis does not take into account individual differences in the EOMR of our participants (see Table S2 in the Supplementary Material) and therefore does not directly address the question of whether the EOMR affects search. To address this issue, we conducted a second analysis in which we compared search times for targets appearing within and above each participant’s individual EOMR.

Array location was recoded so that the EOMR was set to a value of zero. The array position falling just within the EMOR was recoded as location −1. The two array positions falling beyond the EOMR were recoded as locations +1 and +2. For example, for an individual with an EOMR of 32°, the array presented at 29° was be recoded as −1, the array presented at 35° was recoded as +1, the array presented at 38° was recoded as +2. Individual EOMR and the corresponding array locations for within/beyond conditions are reported in Table S2 in the Supplementary Materials.

**Fig. 5:**
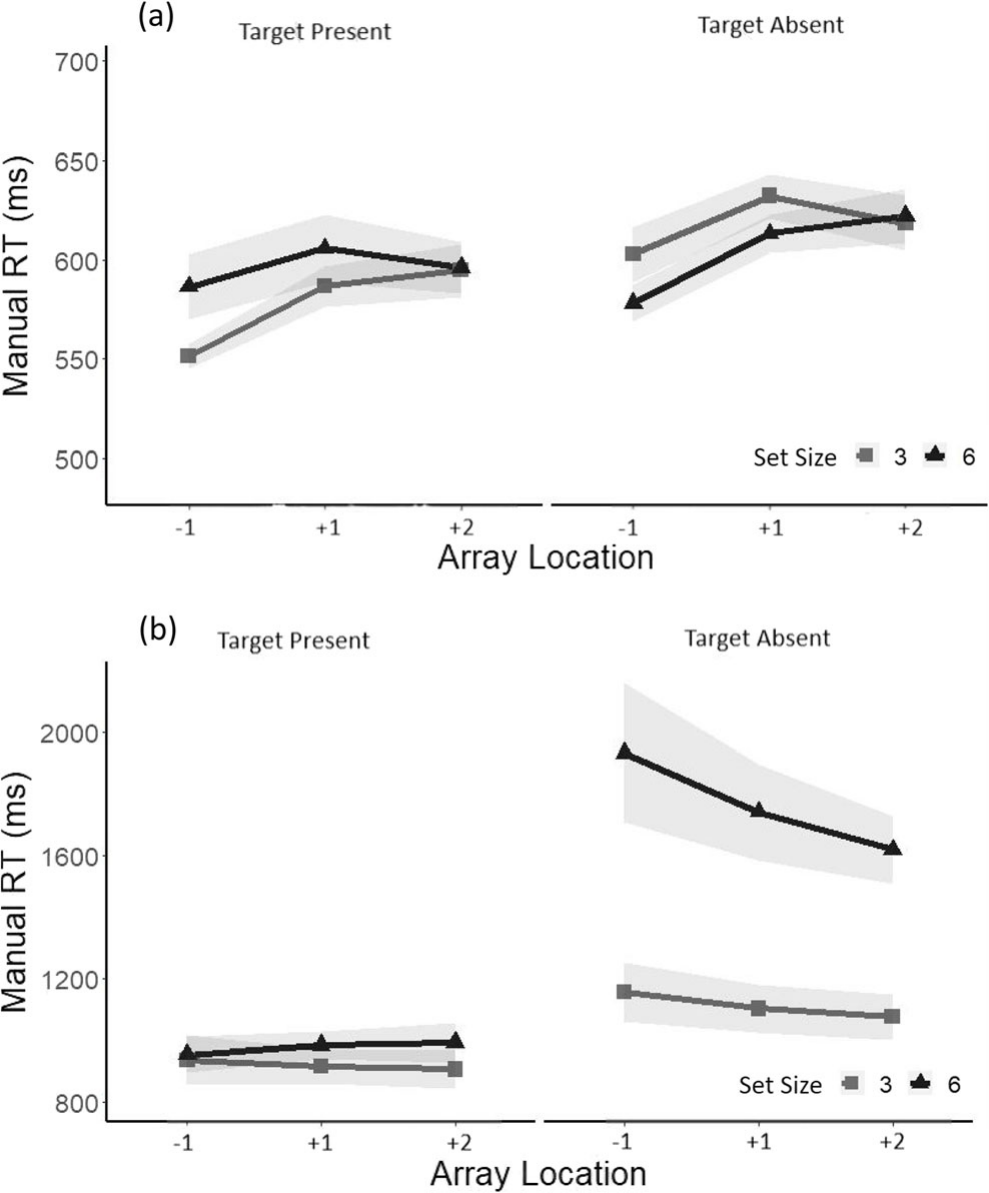
**a** Median reaction times in the Feature search for correct responses in target-present trials (left) and target-absent trials (right) as a function of array location recoded according to each individual’s EOMR and separately for a set size of three (light grey) and a set size of six (dark grey). −1 refers to below EOMR, and +1 and +2 refers to beyond EOMR locations. Shadows represents ±1 *SEM*. **b** Median reaction times in the conjunction search on target-present trials (left) and target-absent trials (right) for correct responses as a function of array location recoded according to each individual’s EOMR and separately for a set size of three (light grey) and a set size of six (dark grey). −1 refers to below EOMR, and +1 and +2 refers to beyond EOMR locations. Shadows represent ±1 *SEM*

The RTs for correct responses in target-present trials were analyzed using a repeated-measures ANOVA, with the factors array location (−1, +1, +2) and set size (3 or 6) at each level of the search task. For the feature search task, there was a significant effect of array location, *F*(2, 28) = 4.20, *p* < .05, ηp_푝_^2^ = .23 (see Fig. 5a). Paired-samples *t* tests revealed that the difference in RTs was significant between the −1/within EOMR (*Mdn* = 568 ms) and the +1/beyond EOMR condition (*Mdn* = 596), *t*(28) = 2.55, *p* = .016, but was not significant between the +1/beyond EOMR and the +2/beyond EOMR condition (*Mdn* = 595), *t*(28) = .0.78, *p* = .93. An ANOVA revealed no significant effect of array size or array location in the conjunction search task (all *F*s ≤ 2.25).

ANOVAs were then performed on RTs for correct responses in the target-absent trials at each level of the search task. In the feature search task, analysis revealed a main effect of array location, *F*(2, 28) = 6.79, *p* < .01, ηp^2^ = .32, such that the difference in RTs was significant solely between the −1/within (*Mdn* = 590 ms) and the +1/beyond (*Mdn* = 622 ms), *t*(28) = 3.29, *p* = .0026. There was no effect of set size and no significant interaction between the two factors (all *F*s ≤ 1.54; see Fig. 5a). In the conjunction search, an ANOVA revealed a main effect of set size (Set Size 3 = 1109 ms; Set Size 6 = 1764 ms), *F*(1, 14) = 10.64, *p* < .01, ηp^2^ = .43, but there was no significant effect of array location, *F*(2, 28) = 3.05, and no interaction, *F*(2, 28) = .92.

#### Response accuracy

Average hit rates and false alarms (FA) are presented on Table 2. As for Experiment 1, participants showed higher hit rates and lower false alarms in the feature colour search task (*M*_Hit_ = .97; *M*_FA_ = .02) compared with the conjunction search task (*M*_Hit_ = .85; *M*_FA_ = .07).

**Table 2:** Average rates and standard deviations for hit, false alarm (FA), and *d* prime (*d'*) separately for each array position in degrees of visual angle (**a–b**) and for each location relative to the EOMR (i.e., −1 vs +1 vs +2; **c**) and each set size

	Feature colour											
**a**	29	29	32	32	35	35	38	38	41	41	44	44
	3	6	3	6	3	6	3	6	3	6	3	6
Hit	.98	.95	.98	.96	.98	.96	.98	.96	.98	.96	.98	.96
	*.02*	*.08*	*.03*	*.08*	*.01*	*.06*	*.02*	*.08*	*.03*	*.07*	*.01*	*.05*
FA	.01	.02	.01	.02	.01	.02	.01	.02	.01	.02	.01	.01
	*.01*	*.06*	*.02*	*.04*	*.01*	*.04*	*.01*	*.05*	*.01*	*.05*	*.01*	*.04*
	Conjunction											
**b**	29	29	32	32	35	35	38	38	41	41	44	44
	3	6	3	6	3	6	3	6	3	6	3	6
Hit	.91	.82	.83	.85	.88	.81	.88	.85	.84	.83	.84	.80
	*.08*	*.10*	*.09*	*.10*	*.08*	*.10*	*.11*	*.07*	*.09*	*.10*	*.12*	*.12*
FA	.02	.14	.04	.1	.04	.12	.06	.09	.06	.09	.06	.1
	*.02*	*.11*	*.04*	*.09*	*.04*	*.09*	*.09*	*.07*	*.08*	*.07*	*.1*	*.08*
	Feature Colour						Conjunction					
**c**	−1	−1	+1	+1	+2	+2	−1	−1	+1	+1	+2	+2
	3	6	3	6	3	6	3	6	3	6	3	6
Hit	.98	.96	.98	.96	.98	.96	.91	.81	.85	.85	.88	.82
	*.02*	*.06*	*.02*	*.08*	*.01*	*.06*	*.08*	*.09*	*.09*	*.11*	*.08*	*.09*
FA	.01	.02	.01	.02	.01	.02	.02	.13	.06	.1	.04	.1
	*.01*	*.04*	*.01*	*.05*	*.01*	*.04*	*.02*	*.1*	*.08*	*.08*	*.05*	*.07*

**Fig. 6:**
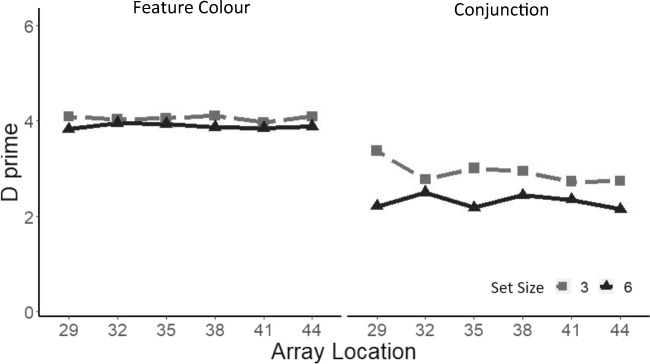
Sensitivity measure (*d* prime) as a function of array location separately for each set size and search task. Shadows represent ±1 *SEM*

Perceptual sensitivity was computed (*d* prime), and an ANOVA performed on *d* prime values showed a main effect of search task, *F*(1, 14) = 120.31, *p* < .0001, ηp^2^ = .89, as sensitivity was significantly higher in the colour search task (*M* = 3.9) compared with the conjunction search task (*M* = 2.6). Participants were also significantly better at detecting the target presence/target absence for a set size of three items (*M* = 3.5) compared with a set size of six items (*M* = 3.1), *F*(1, 14) = 13.43, *p* < .001, ηp^2^ = .49 (see Fig. 6). We also observed a significant interaction between set size and array location, *F*(5, 70) = 3.01, *p* < .05, ηp^2^ = .17. In a one-way ANOVA, with a factor of array location, at each level of the set size revealed that array location had a significant effect on sensitivity for a set size of three, *F*(5, 70) = 3.48, *p* < .01, ηp^2^ = .19, but not for a set size of six, *F*(5, 70) = 1.36. Post hoc *t* tests indicated that this effect was caused by a significant difference between the 29° and the 32° conditions (29 = 3.73, 32 = 3.4), *t*(70) = 3.15, *p* = .0024. All the other comparisons were not significant (all *p*s ≥ .09).

**Fig. 7:**
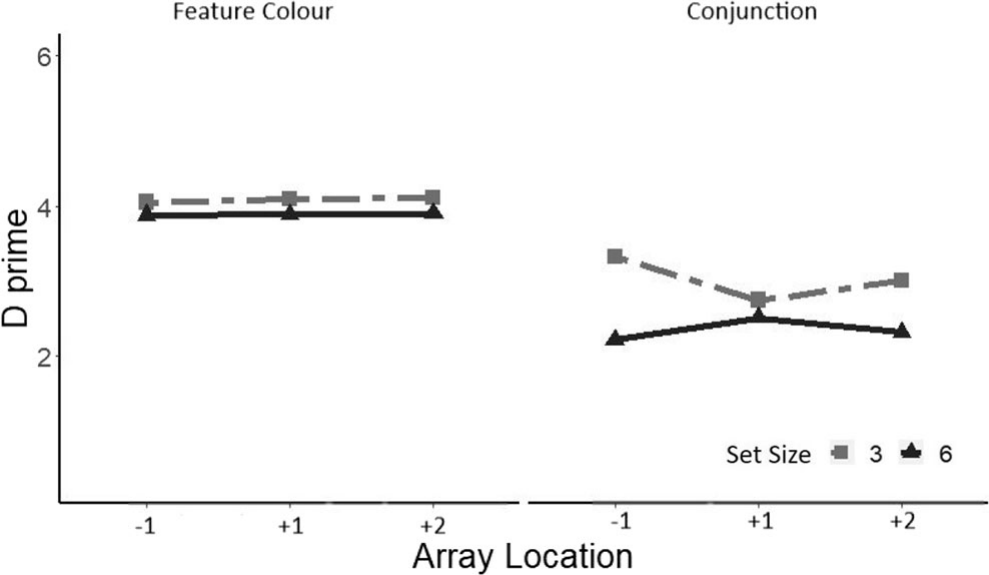
Sensitivity measure (*d* prime) as a function of array location recoded according to each individual’s EOMR, separately for each set size and search task. Shadows represent ±1 *SEM*

We then computed *d* prime as a function of the array location relative to the EOMR (i.e., −1 vs +1 vs +2; see Fig. 7). An ANOVA revealed a main effect of search task, *F*(1, 14) = 127.4, *p* < .0001, ηp^2^ = .90, as well as a main effect of set size, *F*(1, 14) = 6.97, *p* < .001, ηp^2^ = .55. We also observed a significant interaction between set size and array location size, *F*(2, 28) = 3.67, *p* < .05, ηp^2^ = .20. A one-way ANOVA, with a factor of array location at each level of set-size revealed that array location had a significant effect on sensitivity solely for a set size of three, *F*(2, 28) = 4.58, *p* < .05, ηp^2^ = .25. Planned comparisons showed that the difference in sensitivity was only significant between the below (−1: 3.7) and the first beyond location (+1: 3.4) conditions, *t*(70) = 3.03, *p* = .014.

### Discussion

Experiment 2 was designed to address some methodological limitations for Experiment 1 by (a) using a greater range of eccentricities to control for any underestimate in our correction for cortical magnification, (b) making the identity of the feature search target unpredictable to prevent participants implementing search templates, and (c) having equal numbers of target-present and target-absent trials. The results replicated our previous findings that feature search was disrupted when arrays appear beyond the EOMR, that this disruption was observed on both target-present and target-absent trials, and that conjunction search was largely unaffected by the EOMR. Importantly, we are also able to rule out two possible alternative explanations for the results of Experiment 1. Firstly, because the target in the feature search was unpredictable participants were unable to use an attentional template. We can therefore be confident that the slower RTs observed when the arrays were presented beyond the EOMR were due to problems with exogenous orienting, rather than to problems implementing a search template. Secondly, feature search times did not increase monotonically with eccentricity. Rather, they increased steeply when the arrays were presented just beyond the EOMR, then remained similar at increasingly eccentric array positions. Finally, even though median RTs were longer for arrays presented beyond the EOMR, perceptual sensitivity was not affected. Hence, the shorter RTs observed for array presented within the EOMR were not due to a speed–accuracy trade-off effect.

## General discussion

The goal of these studies was to investigate the extent to which feature and conjunction covert visual search were restricted to the eye-movement range. As predicted, the results of Experiment 1 and Experiment 2 showed a clear effect of array location on feature search, such that median reaction times were slower when the array lay beyond the range of eye movements. The objects were scaled according to the cortical magnification factor, conjunction search was not slower beyond the EOMR, and Experiment 2 demonstrated that RTs did not significantly increase with eccentricity once the EOMR had been crossed. It therefore seems very unlikely that the impairment in feature search can be explained solely by differences in the sensory properties of the stimuli. These results are consistent with our previous observations of impaired covert feature search beyond the EOMR (Smith & Archibald, 2018; Smith et al., 2014, Experiment 1; Smith et al., 2010), and a number of studies demonstrating that exogenous covert orienting is impaired beyond the EOMR (Casteau & Smith 2019; Smith et al., 2010; Smith et al., 2012; Smith et al., 2014; although also see Hanning, Szinte, & Deubel, 2019). Together, these studies are evidence that placing a salient target beyond the range of eye movements reduces the efficiency with which it can reflexively summon attention.

The results from the conjunction search task were not quite so clear cut. We observed no significant increase in search times when the array was presented beyond the EOMR, which on first inspection appears consistent with our previous observation that conjunction search is not constrained by the EOMR (Smith et al., 2014, Experiment 2). However, there are a couple of reasons to be cautious when interpreting this result. Firstly, the RTs in the conjunction search tasks were significantly longer and more variable than those in the feature search tasks, which makes the null result a bit harder to interpret. Secondly, although we found a large and significant effect of set size in the target-absent trials, the set size effect on target-present trials was much smaller and not statistically significant. While the set-size effect in target-absent trials is consistent with participants engaging in a serial, attentive search (Chun & Wolfe, 1996) it is not clear why the set size effect in the target-present trials was not statistically significant. One possibility is that because participants knew the colour of the target, they were able to implement a guided search strategy that allowed them to filter out some distractors based on colour, thus making the search more efficient (Smith & Archibald, 2019). Alternatively, the relatively difficult nature of the task may have resulted in some participants adopting idiosyncratic strategies to complete the search. Indeed, although most of our participants showed increased RTs with the number of distractors, three of them appeared to be faster in the set size of six items compared with the set size of three when array was presented beyond the EOMR (see the Supplementary Material). Notwithstanding these caveats, the results of Experiments 1 and 2 seem consistent with the idea that the exogenous attentional processes engaged during feature search are more tightly coupled to the oculomotor system than the more endogenous attentional processes engaged during conjunction search.

At a neural level, the relationship between the oculomotor system and visual search may be best understood in terms of a biased competition account of visual search. In this view, signals relating to the physical properties of the stimulus (e.g., their brightness, size, contrast, orientation) compete in a winner-takes-all competition. This competition occurs in a topographic map of space, called a salience map (Desimone, 1998). Competition in the salience map is influenced by the current goals of the observer, such that the locations of stimuli that possess a feature known to be relevant to the current task are prioritized. In this way the observer can bias the competition towards the stimuli that are most likely to be behaviourally relevant. Fecteau and others have argued that because biased competition relies on integrating low-level stimulus salience with top-down modulations, the map that represents the competition should be referred to a as a priority map rather than a salience map (Fecteau & Munoz, 2006). Bisley and colleagues have convincingly argued that the priority map is instantiated in the lateral intraparietal cortex (Bisley & Goldberg, 2010; Goldberg, Bisley, Powell, Gottlieb, & Kusunoki, 2002), as neural activation in this area correlates with both the onset of saccadic eye movements and the covert selection of targets during visual search (Thomas & Pare, 2007). This region has reciprocal connections with early visual areas, which allows for the modulation of visual signals that characterize visuospatial attention (e.g., Hillyard, Vogel, & Luck, 1998; Luck, Chelazzi, Hillyard, & Desimone, 1997), and with key oculomotor centres such as the frontal eye field (FEF), which is critical for the generation of endogenous orienting signals, and the superior colliculus (SC), which integrates visual and motor signals from cortical and subcortical pathways to specify a saccade goal (Munoz & Everling, 2004; White & Munoz, 2011). In this view, the signal that wins the biased competition can be read out as a covert shift of attention or used by the oculomotor system to guide an overt saccadic eye movement. The oculomotor signals are self-reinforcing, such that activation in the oculomotor system is fed back into the priority map, further biasing activity in favour of the activated location (Bisley, Mirpour, Arcizet, & Ong, 2011). Thus, it seems likely that in addition to salience and goals, activation in the priority map is also influenced by signals from the oculomotor system.

This interaction between stimulus driven signals about physical salience and oculomotor activity relating to potential saccade endpoints can help our understanding of the results of the current study. When a participant must locate a salient target (e.g., in the feature search task), competitive interactions in the priority map rapidly converge to select a single location, which is then powerfully reinforced by the reentrant activation from the oculomotor system (Barash, Bracewell, Fogassi, Gnadt, & Andersen, 1991). This feedback loop allows the visual system to very rapidly and efficiently select the location of the unique feature, irrespective of the number of distractors. This rapid selection, driven by the integration of salience with oculomotor signals, is largely stimulus driven and corresponds to ‘preattentive’ search. Such a selection process is very efficient for locations that can be reached with a saccade. However, stimulus-driven saccades systematically undershoot the intended goal by ~10% of the intended amplitude (Frost & Pöppel, 1976; Stahl, 1999), and this undershoot reflects a suboptimal sensorimotor transformation which is a hardwired property of the oculomotor system (Vitu, Casteau, Adeli, Zelinsky, & Castet, 2017). As a consequence, when stimuli are presented beyond the range of eye movement, there is a discrepancy between the representation of the target location in the SC and the representation of the target location in the rest of the visual system. This will lead to a competition that may end up with selection of the location represented in the oculomotor system rather than the actual target location, thus impeding target detection, exactly as we observed in our experiments.

In contrast, when the target has relatively low salience, as was the case in the conjunction search task, there may be multiple peaks of activation in the priority map, and the competition between the signals takes time to resolve. This, in turn, reduces the capacity for the oculomotor system to influence the competition, as the reinforcing signal from the oculomotor system is distributed across multiple locations. Instead, the competition is resolved by top-down processes that reflect strategic and conscious decisions about which potential target locations should be selected. These processes lead to ‘attentive’ search that is not reliant on activation in the oculomotor system, and therefore not constrained by the EOMR.

Note that many regions known to be involved in saccadic eye movements such as the FEF or the SC are also involved in combined eye–head movements. Several studies shown that a single gaze controller is responsible for programming both the eye and the head (e.g., Khan, Blohm, McPeek, & Lefevre, 2009). In this regard, proponents of OMRH/premotor theory might argue that the ability to prepare for a combined eye–head movement could have still mediate attention shifts (e.g., Cicchini, Valsecchi, & de’Sperati, 2008). This is theoretically plausible; however, there is currently no evidence that preparing, but not executing, a head movement is sufficient to produce a shift of attention. Furthermore, given the evidence that exogenous attention is associated with activation of the head-movement system (Corneil, Munoz, Chapman, Admans, & Cushing, 2008), it is still not clear why the preparation of head movements should support endogenous orienting, but not exogenous orienting.

Our primary goal was to test the idea that preattentive search is constrained by the EOMR. However, our data also have relevance to the debate concerning the functional role of exogenous and endogenous orienting in visual search. More specifically, Briand and Klein (1987) and Briand (1998) have argued that exogenous orienting is required for feature binding in conjunction searches, based on the observation that valid peripheral cues produce larger effects on conjunction search than feature search. In contrast, Kawahara and Miyatani (2001) reported greater facilitation for endogenous cues and Henderickx, Maetens and Soetens (2010) found that exogenous and endogenous cueing produced similar effects on search tasks. If, as Klein and Briand have argued, exogenous attention is required for feature binding, and exogenous orienting is disrupted beyond the EOMR, we should observe a deficit of feature binding beyond the EOMR. Our current studies cannot speak directly to this prediction, but one manifestation of defective feature binding is the presence of illusory conjunctions, which would should to an increased false-alarm rate. In Experiment 1 we observed significantly increased FA rate in the conjunction search task for arrays presented beyond the EOMR, which seems consistent with the views of Briand and Klein (1987).

To briefly summarize, we have argued that the mechanisms involved in preattentive and attentive search are similar to those involved in the exogenous and endogenous modes of spatial attention identified by Posner, Snyder, and Davidson (1980). Exogenous orienting is known to rely on activation of the oculomotor system (Smith et al., 2012), so if preattentive search relies on the same mechanisms as exogenous orienting, it should be subject to the same constraints as covert exogenous orienting. Consistent with this idea, we found that presenting feature search arrays beyond the range of eye movements significantly reduced the efficiency of stimulus-driven preattentive search. This manipulation had no effect on conjunction search, although we speculated that it may have also reduced the efficacy of guided search. This result is consistent with the oculomotor readiness theory of exogenous orienting (Casteau & Smith 2019), and we propose that the relationship between the oculomotor system and visual attention is best understood in terms of a biased competition model of attention in which activation in the oculomotor system biases selection towards the potential goal of an eye movement.

## Electronic supplementary material

ESM 1 (PDF 771 kb)
