# Supplementary material for: On the link between attentional search and the oculomotor system: Is preattentive search restricted to the range of eye movements?
Source: Atten Percept Psychophys. 2020 Jan 15;82(2):518–32. doi: 10.3758/s13414-019-01949-4 (PMC7246251; doi:10.3758/s13414-019-01949-4)
Supplement: Supplementary file 1 — (PDF 771 kb) [file 13414_2019_1949_MOESM1_ESM.pdf]

Supplementary Material

Establishing the Effective Oculomotor Range

Each individual that took part in Experiment 1 (n = 12) and Experiment 2 (n = 15) completed a simple goal directed saccade task in order to measure their effective oculomotor range (EOMR). During this first phase, that lasted about 30 minutes, participants were presented with a discrimination target that could appear at different angular positions on the horizontal axis, always in the temporal hemifield. Participants performed the task monocularly with their dominant eye.

Method

Apparatus

Eye movements of the dominant eye were recorded using a head-mounted EyeLink II (SR Research Ltd., Mississauga, Ontario) at a sample rate of 500 Hz. Because of the large range of eccentricities and the specifications of the eye-tracker, eye-movements were recorded in pupil only mode. Stimuli were generated using PsychoPy (Pierce, 2009) and saved as a jpeg. They were displayed on a 32 inches LED monitor (BenQ) driven by an NVIDIA GeForce GTX 750 Ti graphics board at a refresh rate of 60 Hz. The resolution of the monitor was set at 2560 × 1440 pixels, which corresponded to physical dimensions of 708 mm wide by 398 mm high. At a viewing distance of 30 cm, the display occupied a viewing area of 99° horizontally and 67° vertically.

Material and procedure

Each block of trials started after setting up the eye tracker and running a calibration phase. During calibration, five dots were presented successively on the screen, three dots on the horizontal axis (i.e., (x_{dot1} = 1280 pixels, y_{dot1} = 720 pixels), (x_{dot2} = 905 pixels, y_{dot2} = 720 pixels), (x_{dot3} = 1655 pixels, y_{dot3} = 720 pixels)) and two dots on the vertical midline ((x_{dot4} = 1280 pixels, y_{dot4} = 470 pixels), (x_{dot5} = 1280 pixels , y_{dot5} = 970 pixels)).

Participants were asked to fixate very precisely at each dot location, if fixations were correctly aligned with the calibration dots experiment was started. Otherwise a new calibration phase was initiated.

A drift correction was performed at the beginning of each trial; procedure is illustrated on Figure 1. A fixation stimulus was initially displayed on the computer screen; this consisted of a fixation cross on a black background (.20° x .20°). After a random time interval (400 to 1200 ms), the central cross was removed and the target was displayed (gap of 0 ms).

The target was a circle shape (diameter .20°) filled with black and grey gradient diagonal stripes orientated either leftward or rightward. The target was randomly presented at six various possible eccentricities varying between 29° and 44° (Exp.2) spaced by steps of 3°. Participants were asked to fixate the target as quickly and as accurately as possible and perform an orientation discrimination task using a customized response box set with a TTL trigger. After a delay of two seconds, a new trial began. Session was divided in two blocks of 80 trials (10 repetitions per target eccentricity) and each was preceded by a 10 trials practice block.

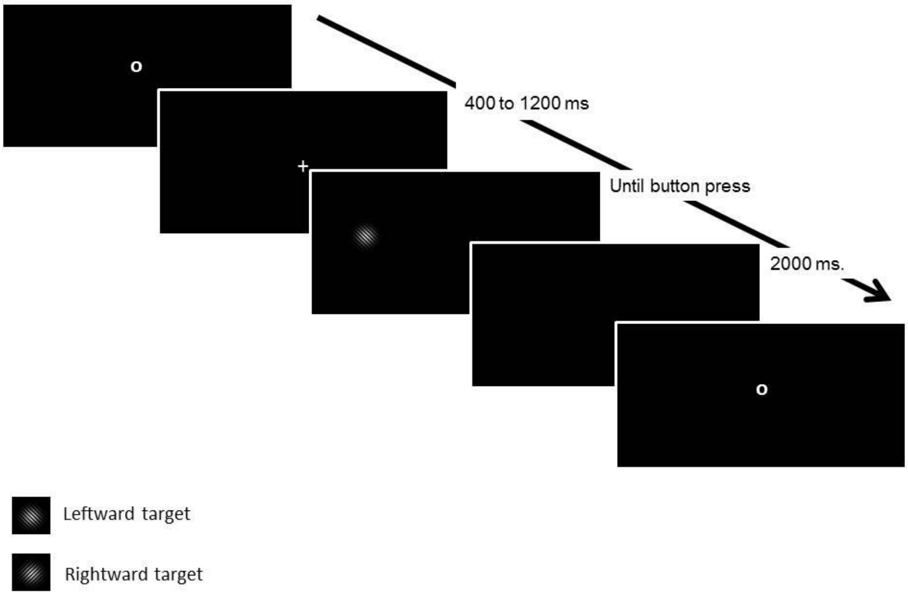

Figure 1. Sequence of events in the EOMR establishment task. Each trial started with a drift correction, followed by a central fixation cross. Target was always presented on the horizontal meridian and participants were instructed fixate as accurately as possible the target and make an orientation discrimination response.

Data selection, results and analyses

Amplitude of the initial saccade following target onset was considered for analysis, this corresponds to the difference between the initial and the final eye position. Each individual data set was analysed separately, however same exclusion criteria were used for all participants. Trials were rejected when (1) first saccade amplitude was of less than 2°, (2) a blink occurred before or after the saccade, (3) the saccade was anticipatory (latency less than 80 ms), (4) initial saccade was in the opposite direction to the target or deviated too much from the horizontal axis and (5) the average eye-position before the saccade deviated from the fixation cross by more than 1° in the horizontal direction.

. *Establishing the Effective Oculomotor Range (EOMR)*

The EMOR was defined as the point at which the mean saccadic gain, defined as the ratio of the actual saccade amplitude to the target eccentricity, dropped to .8 or below. For Exp.1 the participants mean saccade amplitude for targets at this position plus 2 standard deviations

was used to set the target position in the Beyond condition. Their mean saccade amplitude minus 2 SD in the 20° condition was used to set the target position in the Below condition.

Each participant's EOMR are summarized in the table below, separately for Exp.1 and Exp.2.

Table 1.

Individual' EOMR (in degrees) for Exp.1

P1	P2	P3	P4	P5	P6	P7	P8	P9	P10	P11	P12
32	38	37	28	31	34	36	33	31	36	35	34

Table 2.

Eye-movement range, Below and Beyond values in degrees for each of the fifteen participants separately. for Exp.2

	P1	P2	P3	P4	P5	P6	P7	P8	P9	P10	P11	P12	P13	P14	P15
0-*EORM*	32	32	32	35	32	38	38	36	31	32	31	30	32	29	29
-1 *Below*	29	29	29	32	29	35	35	35	29	29	29	29	29	29	29
+1 *Beyond*	35	35	35	38	35	41	41	38	32	35	32	32	35	32	32
+2 *Beyond*	38	38	38	41	38	44	44	41	35	38	35	35	38	35	35

Experiment 1: Results

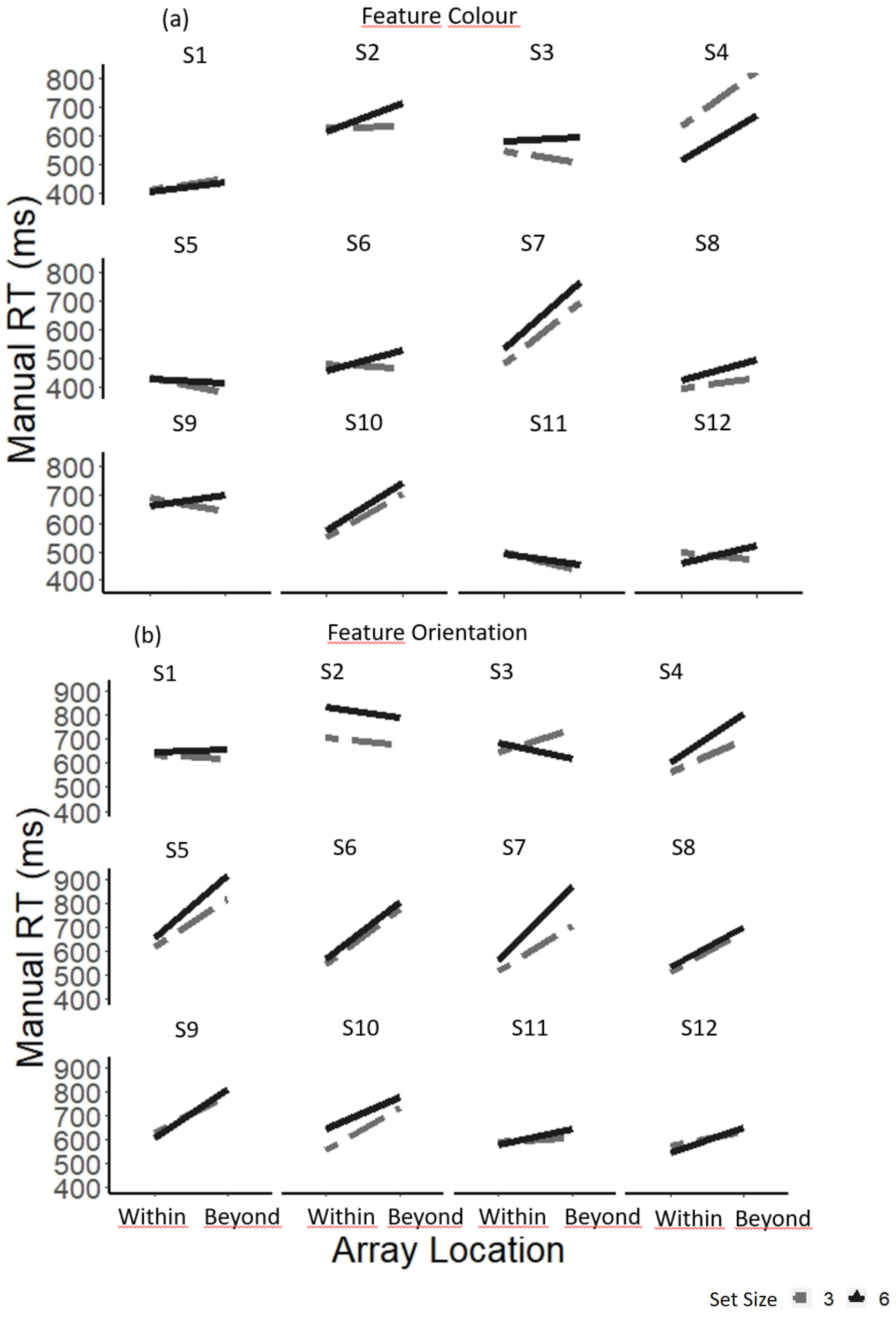

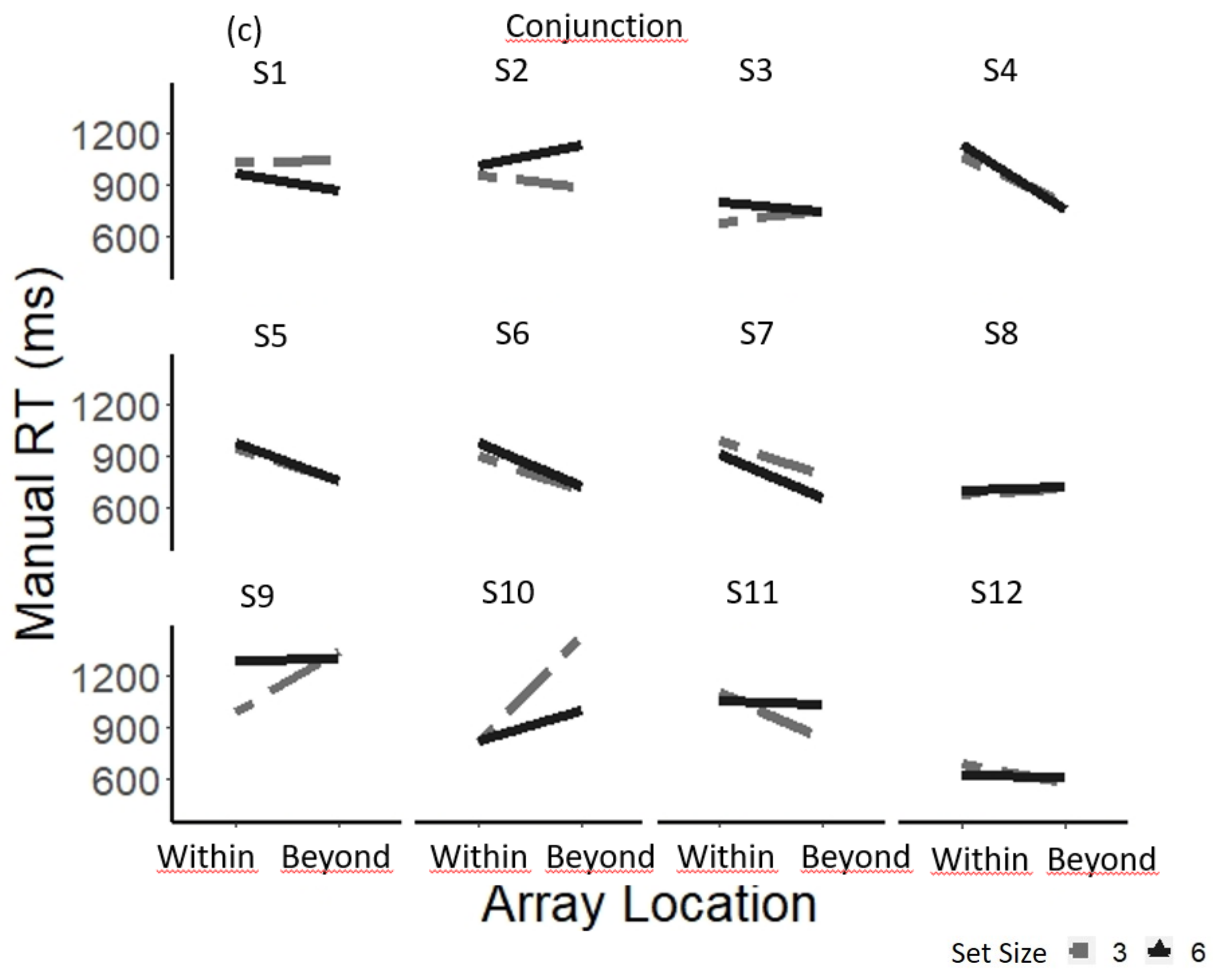

Figure 2. Individual median reaction times for correct responses on target present trials as a function of Array Location and separately for a Set Size of 3 (light grey) and a Set Size of 6 (dark grey) for (a) Feature Colour Search, (b)b Feature Orientation Search, (c) Conjunction Search.

Experiment 2: Results

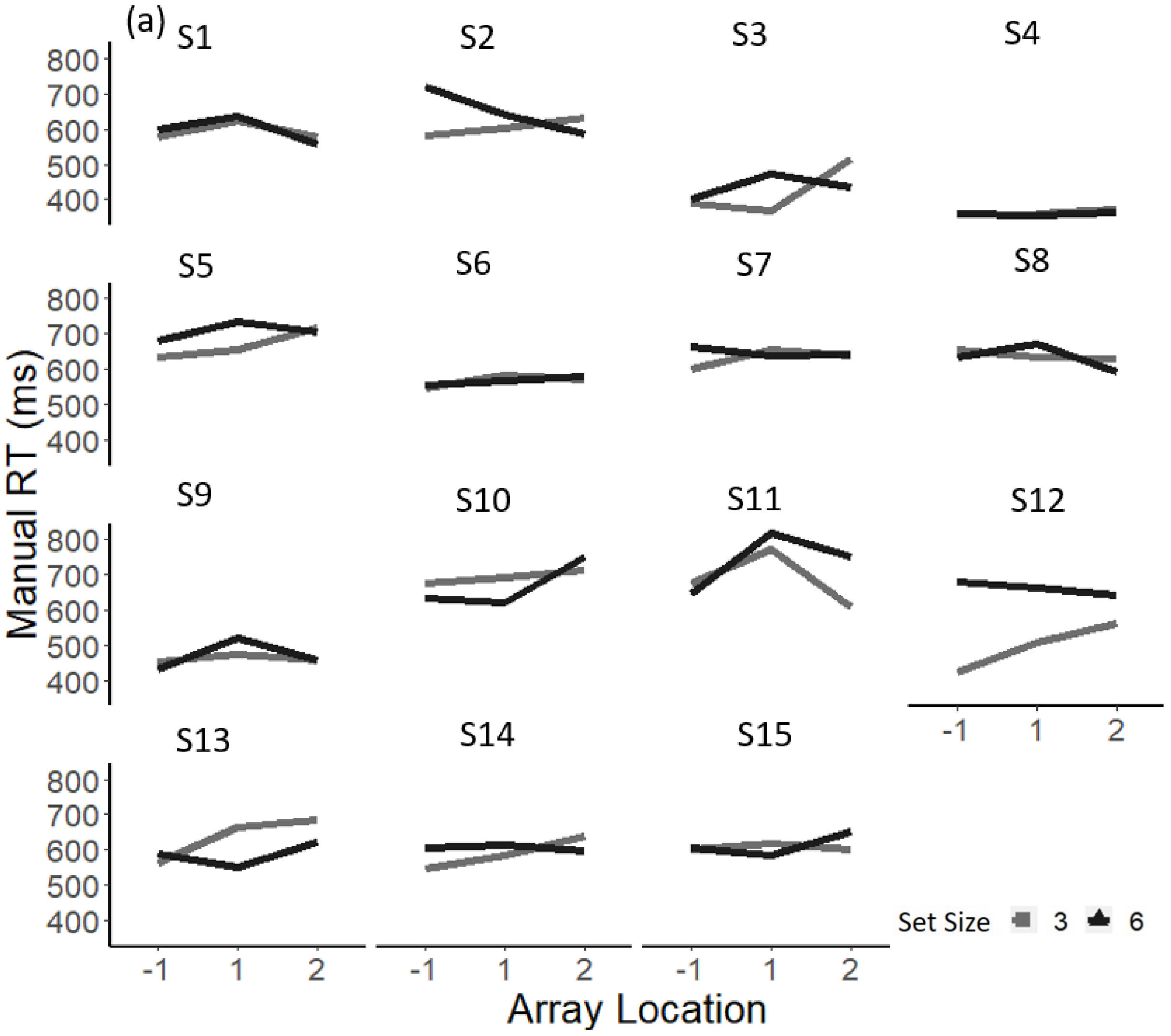

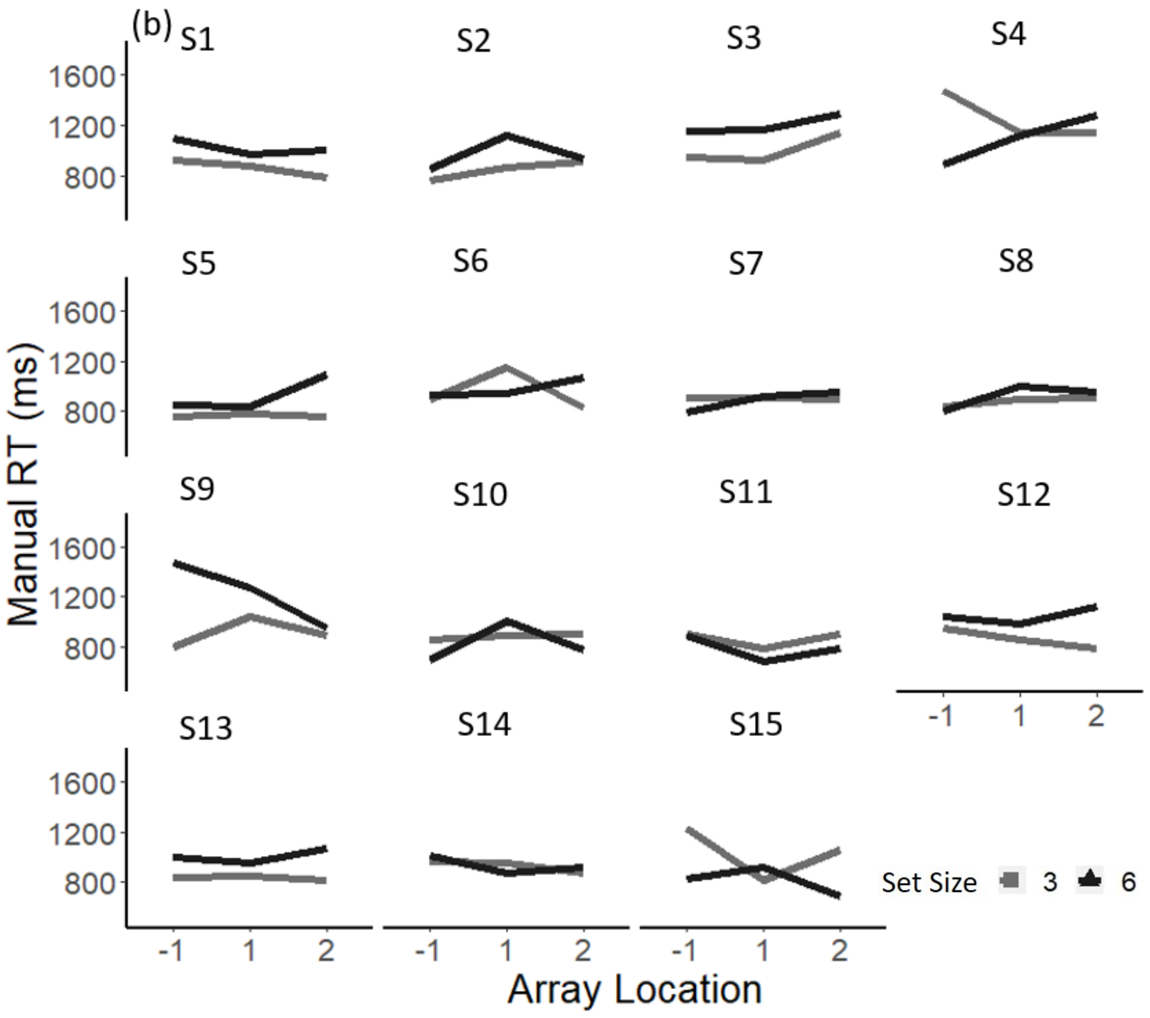

Figure 3 (a-b). Individual median reaction times for correct responses on target present trials as a function of Array Location mapped on each Individual's EOMR and separately for a Set Size of 3 (light grey) and a Set Size of 6 (dark grey) for Feature Colour Search (a) and Conjunction Search (b). -1 refers to Below the EOMR 1 and 2 to locations Beyond the EOMR.
